# Ameliorative Effect of Heat-Killed *Lactiplantibacillus plantarum* Q1 (LPQ1) on Lipid Accumulation: Integration of Network Pharmacology with *In Vitro* and *In Vivo* Experiments

**DOI:** 10.4014/jmb.2503.03038

**Published:** 2025-07-29

**Authors:** Young-Jin Choi, Nishala Erandi Wedamulla, Seok-Hee Kim, Qun Zhang, Duseong Kim, Ji-Won Song, Seunghun Lee, Haeyoung Lee, Jeung Hee An, Ji Young Hwang, Eun-Kyung Kim

**Affiliations:** 1 Jeju Institute of Korean Medicine, Jeju 63309, Republic of Korea; 2 Department of Food Science and Technology, Faculty of Animal Science and Export Agriculture, Uva Wellassa University, Badulla 90000, Sri Lanka; 3 Department of Health Sciences, Graduate School of Dong-A University, Busan 49315, Republic of Korea; 4 Division of Food Bioscience, Konkuk University, Chungju 27478, Republic of Korea; 5 Biohealthcare R&D Center, Hyundai Bioland Co. Ltd., Ansan 15407, Republic of Korea; 6 R&D Center, CUOME BIO Co. Ltd., Jeollanam-do 58141, Republic of Korea; 7 Department of Food and Nutrition, Gangseo University, Seoul 07661, Republic of Korea; 8 Department of Food Science & Technology, College of Enginering, Dong-Eui University, Busan 47340, Republic of Korea; 9 Nutritional Education Major, Graduate School of Education, Dong-A University, Busan 49315, Republic of Korea; 10 Nutrinomics Lab. Co. Ltd., Busan 49315, Republic of Korea

**Keywords:** Obesity, *Lactiplantibacillus plantarum*, paraprobiotics, network pharmacology, inflammation

## Abstract

Lipid metabolism disorders are expanding at an alarming rate worldwide pressuring the entire healthcare system. Therefore, exploring effective therapeutic treatments for lipid metabolism disorders is a hot spot in research. The therapeutic effects of paraprobiotics on obesity are still not fully explored through the integration of network pharmacology analysis with in vitro and in vivo experiments to confirm their beneficial effect. Therefore, the current study aimed to investigate the ameliorative effect of heat-killed *Lactiplantibacillus plantarum* Q1 (LPQ1) on lipid accumulation through the integration of network pharmacology analysis with in vitro and in vivo experiments. Findings of network pharmacology analysis conform with *in vitro* and *in vivo* experiments, revealing that the modulating effect of LPQ1 on lipid metabolism was effective through the regulation of PPAR-γ and TNF-α expression. Therefore, LPQ1 can be promoted as an effective therapeutic agent against lipid metabolism disorders owing to its modulatory effect on hepatic lipid metabolism and inflammation.

## Introduction

Dysregulation of lipid metabolism has been identified as one of the major risk factors for different metabolic problems leading to hyperlipidemia, liver steatosis and hyperglycemia [1]. Growing evidence has suggested an increasing worldwide prevalence of hyperlipidemia which is characterized by accumulated levels of lipids or lipoproteins in the body and is identified as a high-risk factor for atherosclerosis and cardiovascular disease incidence [2]. Elevated levels of triglycerides (TG), total cholesterol (TC), low-density lipoprotein cholesterol (LDL-C) and low levels of high-density lipoprotein cholesterol (HDL-C) are identified as characteristic features of hyperlipidemia [1, 3]. These lipid metabolism disorders are strongly associated with obesity, reporting increased prevalence of obesity globally. Currently, over 40% of males and females have been identified as overweight or obese while drawing much attention towards childhood obesity due to 40 million estimations in the next decade. Long-term intake of fatty foods, unhealthy dietary patterns and a sedentary lifestyle are the major causative factors of overweight and obesity that accelerate the chronic disease risk including nonalcoholic fatty liver disease (NAFLD), diabetes and cancer [4].

NAFLD is the most common chronic liver disease that is characterized by the presence of hepatic steatosis without excessive alcohol consumption. According to current statistics, NAFLD accounts for 34% of global prevalence. Mechanisms which underpin the disease progression of NAFLD are complex and multifactorial [5]. Oxidative stress, inflammation, lipotoxicity, insulin resistance, endoplasmic reticulum stress, mitochondrial dysfunction, gut microbiota and genetic factors are the well-known contributing factors of NAFLD. In NAFLD development, the activities of key regulators such as AMPK, SREBP-1c, PPAR-γ, and TNF-α are interconnected in a way that collectively disrupts hepatic lipid homeostasis [6, 7]. Under energy-deficient conditions, AMPK is activated and phosphorylates ACC, leading to a reduction in malonyl-CoA levels [8]. This not only inhibits fatty acid synthesis but also facilitates mitochondrial fatty acid oxidation. In addition, active AMPK suppresses the transcriptional activity of SREBP-1c, which normally promotes the expression of lipogenic enzymes like FAS, ACC, and SCD1 [7, 9]. Therefore, when AMPK is active, the resultant inhibition of SREBP-1c leads to decreased lipogenesis and lower triglyceride accumulation in hepatocytes. Conversely, when AMPK activity is diminished often due to factors such as inflammation SREBP-1c becomes more active, thereby upregulating lipogenic enzymes and promoting fat synthesis in the liver [10]. Furthermore, proinflammatory cytokines like TNF-α exacerbate this process by inhibiting AMPK activation even further, which intensifies both the activation of SREBP-1c and PPAR-γ [11]. This cascade ultimately leads to increased lipid accumulation and heightened inflammation, driving the progression of NAFLD.

Owing to the complex nature of NAFLD, integrating drug therapy with personalized medicine and lifestyle modification may enhance therapeutic efficacy [12, 13]. On the other hand, effectively managing hyperlipidemia is also equally important in reducing the risk of NAFLD due to their strong association. Furthermore, lipid metabolism in the liver plays a pivotal role in supporting *in vivo* lipid homeostasis as biosynthesis, absorption and catabolism of cholesterol, as well as lipoprotein transport take place in the liver [1]. Therefore, regulating hepatic lipid homeostasis may be a promising approach for managing lipid metabolism disorders. Probiotics are well-defined as effective remedies for lipid metabolism related disorders including NAFLD [14-16]. Although much research has been conducted on the positive effects of probiotics on improving NAFLD, there are several limitations when using live bacteria, such as the risk of infection including sepsis and bacteremia, development of antimicrobial resistance, the potential for toxin secretion, poorly characterized molecular profiles and difficulties in storage and formulation. As an alternative, dead cells, that is, heat-treated paraprobiotics, have recently been attracting attention. Paraprobiotics have the advantage of preserving bioactive components such as cell walls and excellent stability during manufacturing and storage, so they have excellent safety [17]. These paraprobiotics exhibit antitumor, immunoregulatory, anti-inflammatory, and hypocholesterolemic effect [17, 18]. Similarly, the anti-obesity effect of paraprobiotics has been well-defined through several studies proving that the efficacy of paraprobiotics is not directly related to the viability of probiotic cells [19]. The health beneficial properties of paraprobiotics are directly linked to the cell wall components. Peptidoglycans, lipopolysaccharides, teichoic acids, conjugated linoleic acids, β-D-glucans and cell surface proteins have been reported as major bioactive substances of paraprobiotics [18-20].

Recently, the network pharmacology method has gained much attention in investigating the relevant functional targets of different active compounds [21]. Though several studies reported on the ameliorative effect of paraprobiotics on lipid accumulation, integration of network pharmacology with *in vitro* and *in vivo* experiments to investigate the efficacy of paraprobiotics is still not fully explored. Considering this research gap the current study aimed to assess the ameliorative effect of paraprobiotics: *Lactiplantibacillus plantarum* Q1 (LPQ1) on lipid accumulation by integrating network pharmacology with *in vitro* and *in vivo* experiments.

## Methods

### Materials and Reagents

Heat-killed LPQ1 was kindly donated by CUOME BIO Co. Ltd., (Republic of Korea). TRIzol reagent, orlistat, 3-isobutyl-1-methylxanthine (IBMX), dexamethasone, and insulin were procured from Sigma (USA). Fetal bovine serum (FBS) and penicillin-streptomycin antibiotic were purchased from Hyclone (USA) and Gibco (USA), respectively. Serum biochemical analysis kits including total cholesterol (ab285242), low-density lipoprotein cholesterol (ab65390), high-density lipoprotein cholesterol (ab65390), serum triglycerides (ab65336), alanine aminotransferase (ab105134), aspartate aminotransferase (ab105135), leptin (ab199082) and adiponectin (ab108785) were procured from Abcam (England). Anti-AMPK (sc-25792), anti-SREBP-1c (sc-13551), anti-p-ACC (sc-271965), anti-FAS (sc-55580), anti-TNF-α (sc-52746), anti-GAPDH (sc-365062) and anti-β-actin (sc-1616) were purchased from Santa Cruz Biotechnology (USA). Anti-ACC (#3676), anti-PPAR-γ (#2435), anti-C/EBPα (#8178) and anti-p-AMPK (#2535) were purchased from Cell Signaling Technology (USA).

### Preparation of Heat-Killed LPQ1

Heat-killed LPQ1 was prepared following the method described in the previous study with slight modifications [17]. Briefly, LPQ1 was cultured in a medium based on yeast extract, glucose, and soy peptone for 20 h to prepare the heat-killed LPQ1. The cultured LPQ1 was treated at 60-70°C for 2 h to sterilize and then ultrafiltrated. The concentrated LPQ1 was post-sterilized and spray-dried for pulverization.

### Measurement of the Heat-Killed LPQ1 Count

About 1 g of the prepared heat-killed LPQ1 powder with a high concentration was weighed and dissolved in 500 ml of a diluted solution (0.002% citric acid hydrate, 0.1% peptone, 0.85% sodium chloride). The dissolved solution was further diluted up to 5 × 10^4^ using a diluting solution. The diluted solution was observed using a Neubauer counting chamber (Marienfeld-Superior, Germany). The number of cells contained in the 16 small squares was measured by observation at 400 × magnification and the total number of heat-killed LPQ1 was calculated using the formula below.

$$\text{Total number of cells (cells/g) =}\frac{\text{Total number of cells counted in one square}}{\text{Counted surface}\left({\text{mm}}^{\text{2}}\right)\text{×Chamber depth}\left(\text{mm}\right)}{\text{×10}}^{\text{3}}{\text{mm}}^{\text{3}}\text{/g×Dilution factor}$$

### Collection of Targets associated with LPQ1 and Obesity

The Comparative Toxicogenomics Database (CTD; https://ctdbase.org/, accessed on 14 April, 2024) was searched using the keywords “obesity”, “Non-alcoholic Fatty Liver Disease” and “lipid metabolism disorders” to identify the potential targets related to the disease. Then, the online tool Venny 2.0.2 (https://bioinfogp.cnb. csic.es/tools/venny/ index 2.0.2.html, accessed on 14 April 2024) was used to screen the core disease targets. The screened disease targets were used to identify the potential targets for the active compounds of LPQ1 in lipid metabolism disorders.

Next, the STITCH database (http://stitch.embl.de/, accessed on 14 April, 2024) was employed to identify the potential target relevant to active compounds present in paraprobiotics. Subsequently, peptidoglycan was used as the major active compound present in paraprobiotics to construct the compound-target network. The medium confidence cut-off (0.400) was used while limiting the species only to *Homo sapiens*. The Cytoscape software (version 3.10.1) was used to visualize the active compound-target interaction network.

### Construction of Protein-Protein Interaction (PPI) Network

Potential targets of paraprobiotics were overlaid with screened disease targets to identify the potential therapeutic targets of paraprobiotics against lipid metabolism disorders. The online tool Venny 2.0.2 (https://bioinfogp.cnb.csic.es/tools/venny/index2.0.2.html (Accessed on 14 April, 2024) was used to identify the therapeutic targets. The therapeutic targets were imported into the STRING database (https://string-db.org/, accessed on 14 April, 2024) to construct a PPI network (Confidence value > 0.4). The resulting PPI network was visualized using Cytoscape software (version 3.10.1).

### Gene Ontology (GO) and Kyoto Encyclopedia of Genes and Genomes (KEGG) Pathway Enrichment Analyses

The online bioinformatic software ShinyGO 0.80 (http://bioinformatics.sdstate.edu/go/, accessed on 14 August, 2024) was used to perform GO and KEGG pathway enrichment analyses. The false discovery rate (FDR) cutoff was set at 0.05.

### Cell Culture and Palmitic Acid (PA)-Induced Lipid Accumulation in HepG2 Cells

HepG2 cells were cultured in Dulbecco modified eagle’s medium (DMEM) containing 10% FBS and 1%penicillin-streptomycin. Cells were maintained at 37°C in 5% CO_2_ atmosphere. The culture medium was changed every 2 days until the cells reached 80% confluency.

Human hepatocytes were detached with trypsin EDTA solution and seeded in 12-well plates at a density of 1×10^5^ cells/well. The cells in the treatment groups were treated with 0.25 mM of PA and then treated with 50, 100 and 200 μg/ml concentrations of LPQ1 for 24 h. PA was prepared following the method described in the previous study [22]. Following the 24 h treatment period, HepG2 cells were fixed with 10% formalin for 30 min to perform Oil Red O (ORO) staining. After formalin fixation, the cells were washed with PBS three times and then stained with ORO for 30 min. Oil droplets were observed under an optical microscope (Leica, Germany) and captured at × 400 magnification.

Following the treatments HepG2 cells were subjected to total RNA extraction and the extracted RNA was reverse transcribed into cDNA using a cDNA synthesis kit (AccuPower RT PreMix, Bio-Rad, USA). Quantitative PCR was performed with the MIC qPCR cycler (Bio Molecular Systems, Australia), and the relative mRNA expression of sterol regulatory element binding protein-1c (SREBP-1c), fatty acid synthase (FAS) and acetyl-CoA carboxylase 1 (ACC1) were assessed following the 2^−ΔΔCT^ method. Glyceraldehyde-3-phosphate dehydrogenase (GAPDH) was used as an internal reference. The primers used in gene expression analysis are given in Table S1.

### Cell Culture and PA-Induced Lipid Accumulation in FL83B Cells

FL83B cells were cultured in F12K medium supplemented with 10% heat-inactivated FBS and 1% penicillin-streptomycin at 37°C in 5% CO_2_ atmosphere. After reaching 80% confluency FL83B cells were seeded in a complete medium for 24 h and then stimulated with PA (0.25 mM). Then, the cells were treated with concentrations of LPQ1 at 50, 100 and 200 μg/ml for 24 h. The intracellular lipid accumulation was determined by ORO staining as described in section 2.3.1.

### Cell Culture and Palmitic Acid Induced Lipid Accumulation in 3T3-L1 Cells

3T3-L1 preadipocytes were cultured in 10% heat-inactivated bovine calf serum and 1% penicillin-streptomycin containing DMEM medium at 37°C in a 5% CO_2_ atmosphere. 3T3-L1 preadipocytes were maintained at the post-confluent stage for 2 days and differentiation was induced by changing the medium to DMEM supplemented with 10% FBS. The differentiation was induced by adding IBMX (0.5 mM), dexamethasone (1 μM) and insulin (5 μg/ml) as described in a previous study with slight modifications [23]. After 2 days, the medium was switched to DMEM supplemented with 10% FBS and insulin (5 μg/ml). This medium was changed every 2 days and maintained for a total of 4 days. Then, the medium was switched to DMEM supplemented with 10% FBS for 2 days. The sample was treated at concentrations of 50, 100 and 200 μg/ml at each media change. The effect of LPQ1 treatment on adipogenesis was evaluated through ORO staining as described in section 2.3.1.

### Reverse Transcription-Quantitative Polymerase Chain Reaction (RT-qPCR)

Following treatments 3T3-L1 cells were subjected to total RNA extraction using Trizol according to the manufacturer’s instruction. Then, total RNA was reverse transcribed into cDNA with the aid of a cDNA synthesis kit (AccuPower RT PreMix, Bio-Rad). The MIC qPCR cycler (Bio Molecular Systems) was used to perform quantitative PCR, and the relative mRNA expression levels of peroxisome proliferation-activated receptor gamma (PPAR-γ), FAS and tumour necrosis factor alpha (TNF-α) were determined using the 2^−ΔΔCT^ method. GAPDH was used as an internal reference. The primer sequences of the genes used in RT-PCR analysis were given in Table S2.

### Western Blot Analysis

After the treatments, 3T3-L1 cells were washed with PBS and lysed in lysis buffer. Following the quantification of protein, the samples were separated on 12% sodium dodecyl sulfate-polyacrylamide gel electrophoresis (SDS-PAGE) (90 min at 120 V) and transferred onto a nitrocellulose membrane. Non-specific binding was blocked by incubating the membrane with 5% skim milk. Then, the membranes were incubated with specific primary antibodies at 4°C overnight. Following the incubation with peroxidase-conjugated secondary antibodies, the protein bands were detected using an Azure c300 imaging system (Azure Biosystems, USA) [24]. The relative expression level of the proteins was normalized to β-actin.

### Animal Treatment and Sample Collection

Four-week-old male C57BL/6 mice were purchased from Nara Biotech Co., Ltd. (Republic of Korea). The mice were caged in clear plastic crates and maintained under controlled environmental conditions (Temperature: 20-21°C; Relative humidity: 40-45%; 12/12 h dark/light cycle). Feed and water were provided ad libitum. After one week of acclimatization period, the mice were randomly allocated into five treatment groups (n =8). The control group was fed a normal diet, and all the other groups were fed a high-fat-diet (HFD) for 8 weeks to establish obese mouse models. The subsequent treatment groups were as follows: (1) control group (Con): fed a normal diet (fat 10 kcal%) and daily administration of distilled water; (2) high-fat-diet group (HFD): fed a HFD (fat 60 kcal%) and daily administration of distilled water; (3) low dose LPQ1 treatment group (LP-L): fed with HFD and daily administration of 7.767 × 10^9^ cells/kg of LPQ1; (4) high dose LPQ1 treatment group (LP-H): fed with HFD and daily administration of 1.553 × 10^10^ cells/kg of LPQ1; (5) positive control group (Orlistat): fed with HFD and daily administration of orlistat 60 mg/kg. All the treatments were continued for 4 weeks. All the animal tests were performed in accordance with the guidelines for the Care and Use of laboratory animals and approved by the Dong-A University Animal Care and Use Committee (DIACUC-DIACUC-23-40).

After 12 weeks of treatment mice were fasted overnight and sacrificed under anesthesia. Sera were separated from the blood samples collected by cardiac puncture and stored at -80°C for subsequent analysis. The liver, epididymal fat and perirenal fat were immediately excised and weighed. Then, partial samples of excised liver tissues were frozen in liquid nitrogen and stored at -80°C until further analysis. The remaining liver and epididymal tissues were fixed in 10% formaldehyde for histological analysis.

### Body Fat Composition Analysis

Body fat composition of the Con, HFD and treatment groups were compared using dual energy X-ray absorptiometry (DEXA) measurements. DEXA measurements were performed using a total-body scanner (InAlyzer dual X-ray absorptiometry, Medikors, Republic of Korea) after continuing treatments for 4 weeks. Anesthetized mice were located on the scanner bed while stretching the tail and limbs away from the body.

### Biochemical Analysis

The serum concentration of total cholesterol, low-density lipoprotein cholesterol, high-density lipoprotein cholesterol, serum triglycerides, alanine aminotransferase, aspartate aminotransferase, leptin and adiponectin were measured with ELISA kits (Abcam) according to the manufacturer’s instructions.

### Reverse Transcription-Quantitative Polymerase Chain Reaction (RT-qPCR)

RNA was extracted from liver tissues using Trizol in accordance with the instructions given by the manufacturer. Then, first-strand complementary DNA (cDNA) was synthesized using the AccuPower RT PreMix (Bio-Rad) kit for cDNA synthesis. ACC1, FAS, stearoyl-CoA desaturase 1 (SCD1), PPAR-γ, diacylglycerol-acyltransferase (DGAT), 3-hydroxy-3-methylglutaryl-CoA reductase (HMGCR), hormone-sensitive lipase (HSL), and CCAAT/enhancer binding protein alpha (C/EBPα) were estimated by quantitative PCR using the MIC qPCR cycler (Bio Molecular Systems). The mRNA expression level of GAPDH was used as an internal reference. The primer sequences are listed in Table S2. The relative gene expression was expressed using the 2^−ΔΔCT^ method. The PCR conditions were as follows: 95°C (denaturation temperature) for 10 s, 60°C (annealing temperature) for 20 s and 72°C (extension temperature) for 10 s by 40 cycles.

### Histological Analysis

Liver and epididymal fat tissues were fixed in 10% formaldehyde for 24 h. Then dehydrated, embedded and sectioned into 4 μm thickness for staining. Subsequently, the tissue sections were stained with hematoxylin and eosin (H&E) after dewaxing. The stained sections were sealed, and the liver morphology was captured using an optical microscope (Leica, Germany).

### Western Blot Analysis

Frozen liver tissues were homogenized and lysed using RIPA lysis buffer. The samples were centrifuged (15,928 ×*g*) at 4°C for 20 min to collect the protein in the supernatant. The protein samples were quantified using bicinchoninic acid assay and separated on 12% SDS-PAGE (90 min at 120 V) and transferred onto a nitrocellulose membrane. Following the blocking with 5% skim milk for 1h at room temperature, the membrane was incubated with the primary antibody overnight at 4°C. Then, the membrane was incubated with peroxidase conjugated secondary antibodies. Finally, immunoreactive bands were visualized using an Azure c300 imaging system (Azure Biosystems, USA). ImageJ 1.47v software was employed to quantify the chemiluminescent intensity of each protein.

### Immunohistochemistry

Liver tissue sections were deparaffinized and hydrated before antigen retrieval with 0.01 mol/l citrate buffer (pH 6.0) by autoclaving for 7 min at 120°C. Sections were allowed to cool for 30 min at room temperature. Following washing with distilled water, 3% H_2_O_2_ was used to quench the endogenous peroxidase activity. After blocking with normal goat serum, tissue sections were incubated overnight at 4°C with anti-TNF-α and anti-PPAR-γ. Following treatment with the secondary antibody, all the sections were counted and stained with hematoxylin for capture using an optical microscope (Leica).

### Statistical Analysis

All experimental data were expressed as means ± standard deviation. The significant difference between the samples was analyzed using one-way analysis of variance with Dunnett’s post hoc tests. All data was analyzed by GraphPad Prism 9.0 (GraphPad Software Inc., USA), and *p* < 0.05 was considered statistically significant.

## Results

### Network Pharmacology Analysis

**Fig. 1:**
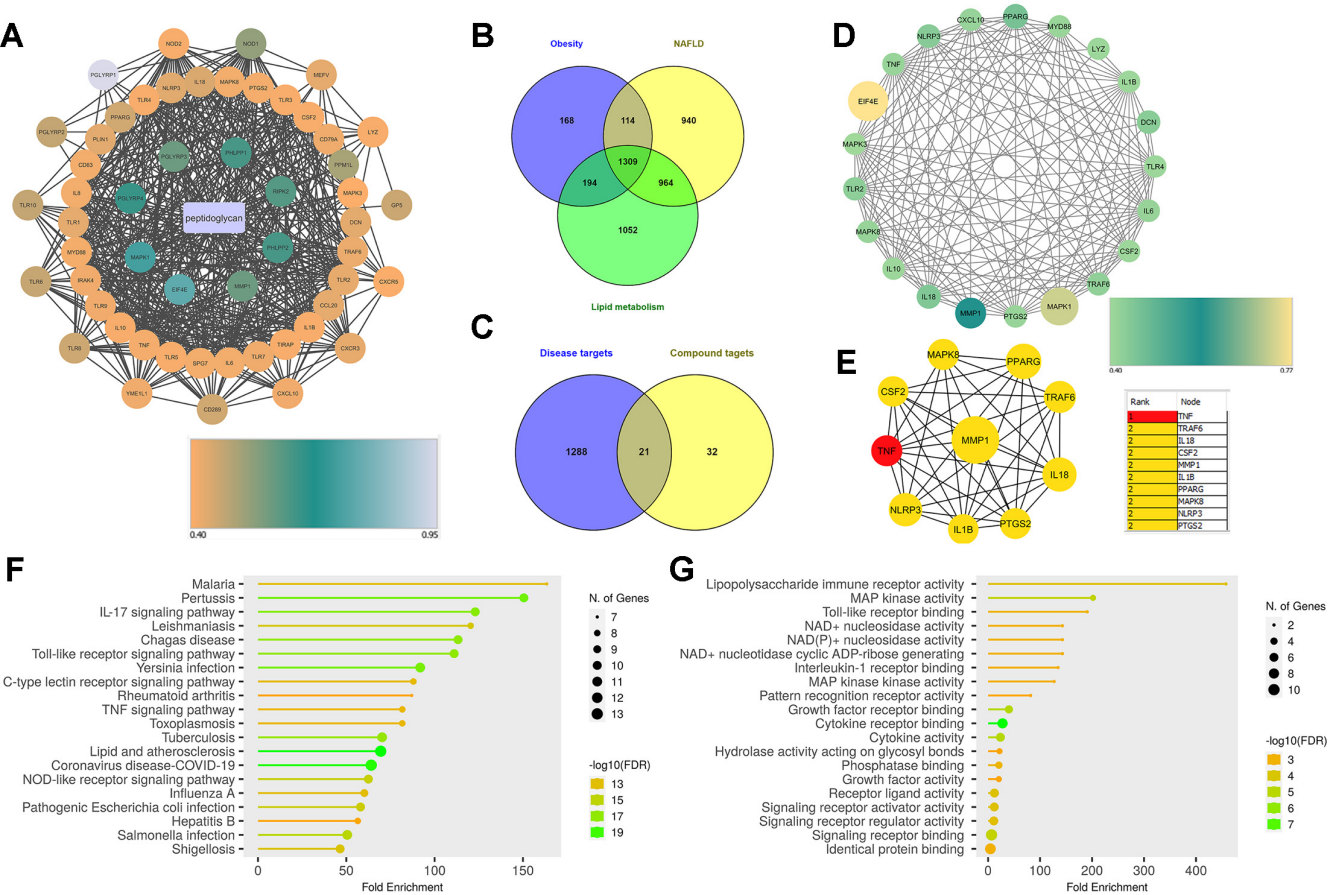
Identification of core targets and pathways of paraprobiotics using network pharmacology analysis. (**A**) The constituent-target protein network constructed by Cytoscape. Varied color of the nodes denoted the STITCH combined score. (**B**) Venn diagram displaying the 1390 common targets for lipid metabolism related diseases: non-alcoholic fatty liver disease, obesity and lipid metabolism disorders. (**C**) Venn diagram screening 21 core targets related to lipid metabolism. (**D**) The PPI network of 21 core targets constructed by Cytoscape. Varied sizes and color intensities of the nodes denoted the STRING score. (**E**) Top 10 hub genes screened from Ec Centricity algorithm of the CytoHubba plugin of Cytoscape. Varied sizes of the nodes denoted the combined score, and varied color intensities denoted the rank. (**F**) KEGG enrichments analysis of 21 core targets of paraprobiotics. (**G**) GO enrichments analysis (molecular function) of 21 core targets of paraprobiotics.

Based on the previous studies, peptidoglycan has been identified as the main bioactive compound present in paraprobiotics which contributing to health beneficial properties [18-20]. Subsequently, 53 protein targets (Table S3) were identified after screening the peptidoglycan in the STITCH database using medium confidence (0.4). The constituent-target protein network was constructed by Cytoscape and displayed in Figure 1A. Different color intensities denote the STITCH combined score.

Differentially expressed disease genes were screened in the comparative toxicogenomics database (Inference Score >60) using three different keywords to identify core disease genes. Subsequently, a total of 1,309 differentially expressed genes (Table S4) were identified as common to lipid metabolism disorders, obesity and NAFLD by Venn diagram (Fig. 1B). Then a total of 21 relevant targets were screened by the Venn diagram (Fig. 1C), which were the candidate targets of paraprobiotics involved in lipid metabolism. The complex interactions of these 21 targets were illustrated in the PPI network and visualized using Cytoscape (Fig. 1D). Different color intensities and sizes of the nodes represented the STRING interaction score. The PPI network contained 21 nodes and 151 edges. The potential targets of paraprobiotics related to lipid metabolism were further analyzed using the CytoHubba plugin of Cytoscape to screen the hub genes. The top ten hub genes selected from the Ec Centricity algorithm of CytoHubba were displayed in Fig. 1E. Different color intensities denote the Ec Centricity rank and different sizes denote the interaction score. TNF-α, TRAF6, IL-18, IL-1β and PPAR-γ were the major hub genes identified.

The results of the KEGG and GO enrichment analysis are shown in Fig. 1F and G, respectively. As displayed in the figure KEGG analysis of potential targets of paraprobiotics in lipid metabolism pinpointed IL-17, toll-like receptor, TNF and NOD-like receptor signaling as associated pathways of paraprobiotics. Further analysis of common factors of the above KEGG enrichment pathways identified nuclear factor kappa B (NF-κB) and its associated genes as the major common factor for most of the pathways. Concurrently, GO enrichment analysis identified lipopolysaccharide immune receptor activity, MAP kinase activity and cytokine activity as major biological processes associated with paraprobiotics in the regulation of lipid metabolism. Therefore, as revealed by the network pharmacology analysis, the regulatory effect of LPQ1 on lipid metabolism is linked with inflammatory responses.

### LPQ1 Modulates Cellular Lipid Metabolism and Inflammatory Responses

**Fig. 2:**
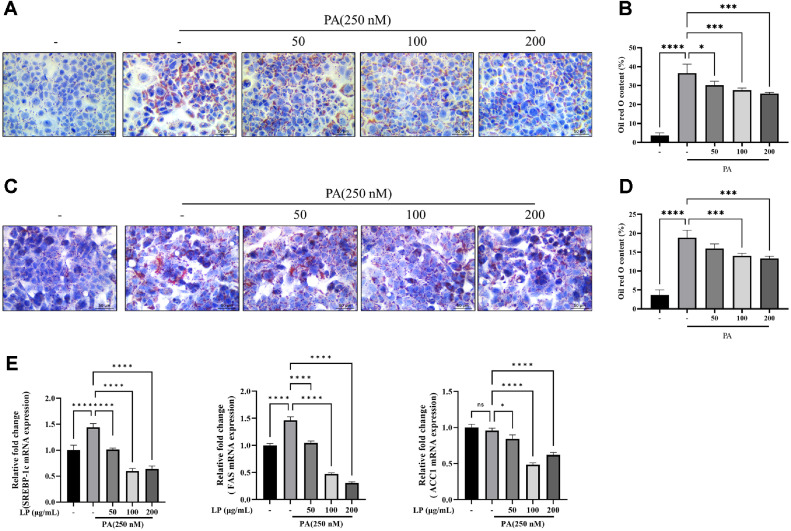
LPQ1 (LP) treatment decreased palmitic acid (PA)-induced lipid accumulation in HepG2 and FL83B cells. (**A**) Oil Red O staining of PA-induced FL83B cells treated with LP. FL83B cells were exposed to 250 μM PA and treated with different concentrations (50, 100, 200 μg/ml) of LP for 24 h. Intracellular lipid accumulation of FL83B cells was visualized by Oil Red O staining and oil droplets were captured by light microscope at 400х magnification. (**B**) Quantification of intracellular lipid accumulation of FL83B cells using ImageJ software, displayed in the histogram as percentage of Oil Red O content. (**C**) Oil Red O staining of PA-induced HepG2 cells treated with LP. HepG2 cells were exposed to 250 μM PA and treated with different concentrations (50, 100, 200 μg/ml) of LP for 24 h. Intracellular lipid accumulation of HepG2 cells was visualized by Oil Red O staining and oil droplets were captured by light microscope at 400х magnification. (**D**) ImageJ-based quantification of intracellular lipid accumulation (% Oil Red O content) in HepG2 cells (**E**) Effect of LP treatment on mRNA levels of major lipid metabolism related genes. The expression of SREBP-1c, FAS and ACC1 was assessed by qPCR with specific primer pairs. All the data were expressed as mean ± SD of three independent experiments. *p < 0.05, ***p < 0.001 and ****p < 0.0001 compared to PA-induced group.

Regulatory effect of LPQ1 on lipid metabolism and inflammatory responses was studied *in vitro* using HepG2, FL83B and 3T3-L1 cell lines. The cellular models were treated with different concentrations of LPQ1: 50, 100, 200 μg/ml to assess the anti-obesity effect. The cell counting results revealed that the LPQ1 contained 1.63 × 10^12^ cells/g. FL83B and HepG2 cells were employed to confirm the ameliorative effect of LPQ1 on hepatic lipid accumulation. Consequently, PA-induced FL83B and HepG2 cells were treated with different concentrations of LPQ1 (50, 100, 200 μg/ml) and intracellular lipid accumulation was visualized with ORO staining. Subsequently, FL83B cells were stimulated with 0.25 mM PA to assess the effectiveness of LPQ1 on cellular lipid metabolism. Intracellular lipid accumulation was determined by ORO staining after 24 h co-treatment of FL83B cells with PA and LPQ1 (50, 100, 200 μg/ml). Intracellular lipid accumulation of PA-stimulated cells was significantly higher than that of the control group and LPQ1 treatment markedly decreased the lipid accumulation in PA-induced FL83B cells in dose-dependent manner (Fig. 2A and 2B). Therefore, LPQ1 treatment effectively suppressed the PA-induced lipid accumulation in FL83B cells. As displayed in Fig. 2C and 2D, PA stimulation markedly increased the lipid accumulation in HepG2 cells compared to that of the control group, as visualized by a dramatic increase in the number of lipid droplets (Fig. 2C). The ORO positive area was quantified using ImageJ software and visualized in Fig. 2D. LPQ1 treatment effectively decreased the PA-induced lipid accumulation compared to the PA-treated model group.

Relative mRNA expression results of LPQ1 treated HepG2 cells confirmed that LPQ1 treatment significantly decreased the relative mRNA expression levels of SREBP-1c, FAS and ACC1 in PA-induced HepG2 cells (Fig. 2E). Concurrently, LPQ1 treatment effectively ameliorates the lipid metabolism related gene expression in PA-induced HepG2 cells.

### LPQ1 Inhibits Adipogenic Differentiation of 3T3-L1 Cells

**Fig. 3:**
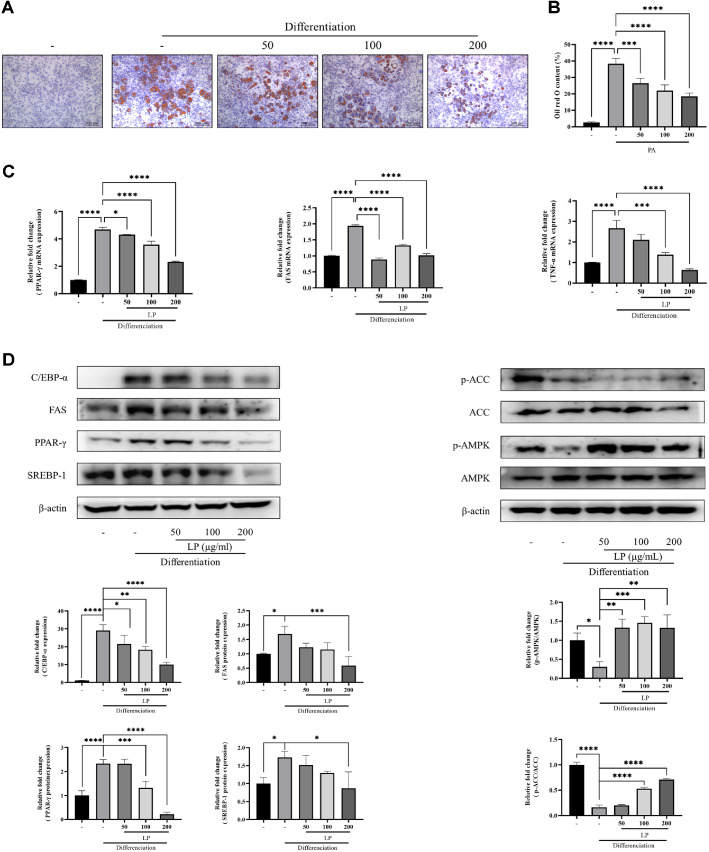
LPQ1 (LP) treatment decreased palmitic acid (PA)-induced lipid accumulation in 3T3-L1 adipocytes. (**A**) Oil Red O staining of 3T3-L1 adipocytes treated with different concentration of LP. 3T3-L1 preadipocytes were incubated in differentiation medium in the presence of LP (50, 100, 200 μg/ml) for 8 days. Lipid droplet formation was visualized with Oil Red O staining and photographed using a light microscope at 400х magnification. (**B**) Quantification of lipid droplet formation of 3T3-L1 adipocytes as a percentage of Oil Red O content. (**C**) Effect of LP treatment on mRNA levels of major adipogenic transcription factors and inflammation related genes in 3T3-L1 adipocytes. 3T3-L1 preadipocytes were incubated in differentiation medium in the presence of LP (50, 100, 200 μg/ml) for 8 days. The expression of PPAR-γ, FAS and TNF-α was assessed by qPCR with specific primer pairs. (**D**) Reduction in the expression of lipid metabolism and inflammation related protein by LP in 3T3-L1 adipocytes. 3T3-L1 preadipocytes were incubated in differentiation medium and treated with different concentrations of LP (50, 100, 200 μg/ml). Total proteins were extracted from cell lysates and protein expression of SREBP-1c, PPAR- γ, FAS, C/EBP-α, p-AMPK and p-ACC were measured by Western blot analysis. β-actin was used as internal control to normalize the intensity of protein bands. The western blot results were quantified and illustrates in the histogram. All the data were expressed as mean ± SD of three independent experiments. *p < 0.05, ***p < 0.001 and ****p < 0.0001 compared to differentiated group.

The ameliorative effect of LPQ1 on lipid accumulation was studied using 3T3-L1 preadipocytes. Preadipocytes were treated with different concentrations (50, 100, 200 μg/ml) of LPQ1 after initiating the differentiation of 3T3-L1 preadipocytes into adipocytes. On day 8 of the treatment cells were fixed with formalin and lipid accumulation was assessed by ORO staining. As revealed by the ORO staining results, the ORO positive area was dose-dependently decreased with the increase in concentration of LPQ1 (Fig. 3A and 3B). Therefore, this confirms the effectiveness of LPQ1 in inhibiting lipid accumulation.

The current study further investigated the mRNA and protein expression levels of PPAR-γ, FAS and TNF-α to confirm the ameliorative effect of LPQ1 on lipid accumulation. The results are displayed in Fig. 3C. As shown in the results LPQ1 treatment downregulated the mRNA expression levels of PPAR-γ, a major regulator of adipocyte differentiation. Similarly, LPQ1 treatment downregulated the mRNA levels of the inflammatory cytokine TNF-α. Consequently, the mRNA expression results are in line with the outcomes of the network pharmacology analysis, where the regulatory effect of LPQ1 on lipid metabolism is mainly linked with the hub genes: PPAR-γ and TNF-α identified in the networking. Moreover, LPQ1 treatment decreased the protein expression levels of PPAR-γ, FAS, SREBP-1c and C/EBP-α while elevating the expression levels of p-ACC and p-AMPK (Fig. 3D). These protein and gene expression results revealed that LPQ1 is effective in inhibiting adipocyte differentiation resulting in reduced levels of intracellular lipid accumulation via concomitant downregulation of PPAR-γ and the inflammatory factor, TNF-α.

### LPQ1 Reduced HFD-Induced Body Weight Gain and Fat Deposition

**Fig. 4:**
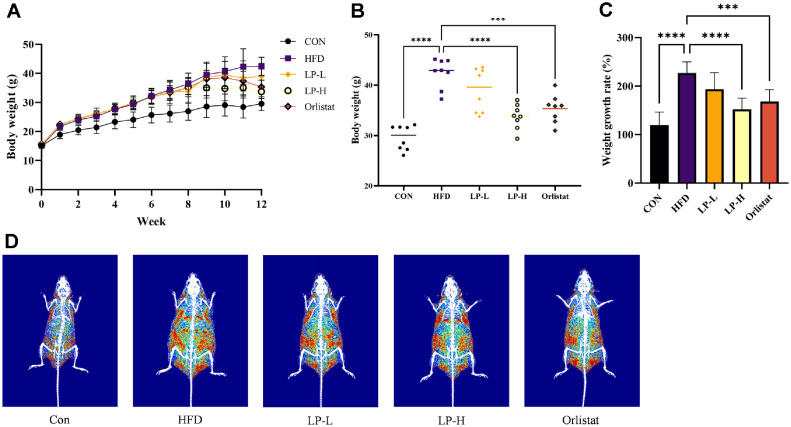
Oral administration of LPQ1 attenuates high-fat diet (HFD)-induced body weight gain in obese C57BL/6 mice. (**A**) Effect of oral administration of LPQ1 on body weight changes during 12-week growth period. (**B**) Body weight of mice measured in the last week. (**C**) The percentage growth rate of body weight (**D**) The representative images of radiograph of each treatment group taken by dual energy X-ray absorptiometry (DEXA). C57BL/6 mice were fed a normal chow diet or HFD for 12 weeks and treated with LPQ1 and orlistat in last 4 weeks. Two oral doses of LPQ1 were daily administered as high (LP-L: 7.767 × 10^9^ cells/kg) and low (LP-H: 1.553 × 10^10^ cells/kg) while positive control group treated with 60 mg/kg orlistat. After 12 weeks of experimental period all mice were sacrificed over anaesthesia and liver samples were collected for further analysis. Con: control group; HFD: high-fat-diet group; LP-L: low dose LPQ1 treatment group; LP-H: high dose LPQ1 treatment group; Orlistat: positive control group. The results expressed as mean ± SD and the significant values are denoted based on the Dunnett's multiple comparisons test. ***p < 0.001 and ****p < 0.0001 compared to HFD group.

Mice were fed with HFD for 8 weeks to establish HFD-induced an obese mice model. After 8 weeks of HFD feeding, the body weight of mice in the HFD, LP-L, LP-H and Orlistat groups was markedly increased compared to the Con group (Fig. 4A). A marked reduction in weight growth rate was observed in the LP-L, LP-H and Orlistat groups after one week of treatment (Fig. 4A). This reduction was more pronounced in the LP-H and Orlistat groups than in the LP-L group. Therefore, LPQ1 is effective in reducing HFD-induced body weight gain. The body weight of mice in the LP-H and Orlistat groups was significantly (*p* < 0.05) lower than that of the HFD group after continuing the oral administration of LPQ1 or orlistat for 4 weeks with HFD feeding (Fig. 4B). The weight growth rate also displayed similar trends where the LP-H and Orlistat groups exhibited significantly lower weight growth rates compared to that of the HFD group (Fig. 4C). The weight growth rate of the LP-H and Orlistat groups was reduced to 152.48% (*p* < 0.0001) and 168.33% (*p* < 0.001), respectively compared to that of HFD group (227.15%). Furthermore, high doses of LPQ1 more effectively decreased the weight growth rate of HFD-induced mice than that of orlistat. Fat density of the entire body was scanned with DEXA to confirm the effectiveness of LPQ1 in decreasing the HFD-induced body weight via an ameliorative effect on fat deposition (Fig. 4D). As displayed in the figure, the HFD group was characterized by an increase in fat density throughout the body and abdominal area compared to the control group. Interestingly, the administration of LPQ1 significantly reduced fat deposition caused by the HFD. Additionally, oral administration of orlistat also markedly decreased the HFD-induced fat deposition.

### LPQ1 Reduced HFD-Induced Lipid Accumulation in Liver and Adipose Tissues

**Fig. 5:**
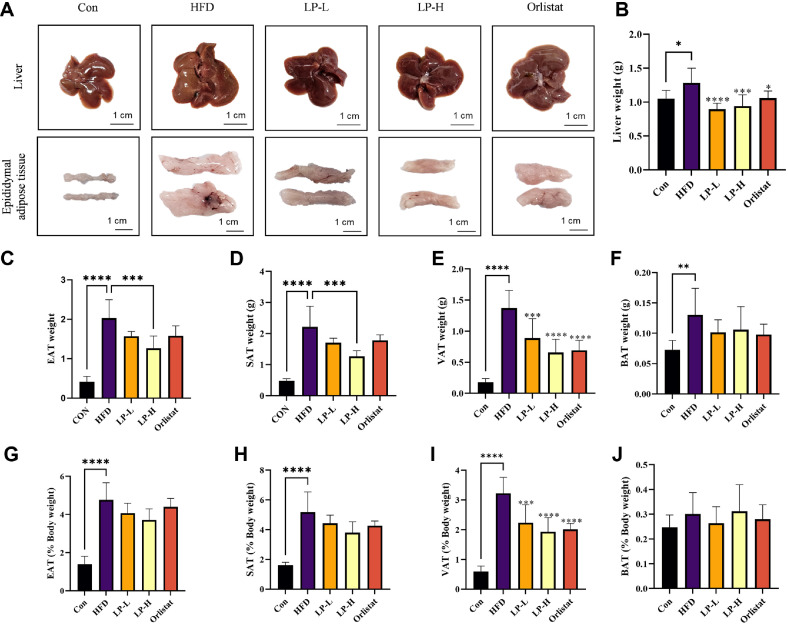
Effect of LPQ1 on the physiological indexes and fat accumulation of obese C57BL/6 mice. (**A**) Morphological characteristics of liver and epididymal adipose tissues. Weight of (**B**) Liver, (**C**) Epididymal adipose tissues (EAT), (**D**) Subcutaneous adipose tissues (SAT), (**E**) Visceral adipose tissues (VAT) and (**F**) Brown adipose tissues (BAT) of each treatment group. Physiological index of (**G**) EAT, (**H**) SAT, (**I**) VAT and (**J**) BAT expressed as percentage of body weight. C57BL/6 mice were fed with normal chow diet or high-fat-diet (HFD)- for 12 weeks and HFD-induced mice were treated with LPQ1 or orlistat in last 4 weeks. After 4 weeks of treatment period mice were sacrificed and liver, epididymal fat and perirenal fat were immediately excised and weighed. Con: control group; HFD: high-fat-diet group; LP-L: low dose LPQ1 treatment group; LP-H: high dose LPQ1 treatment group; Orlistat: positive control group. The results expressed as mean ± SD and the significant values are denoted based on the Dunnett's multiple comparisons test. *p < 0.05, **p < 0.01, ***p < 0.001 and ****p < 0.0001 compared to HFD group.

Different adipose tissues and the liver were excised at the end of the treatment period to investigate the effect of LPQ1 on *in vivo* lipid accumulation. Figure 5 shows the effect of LPQ1 on lipid accumulation in the liver and different adipose tissues. The liver and epididymal adipose tissues were photographed immediately after excising the respective tissues and are shown in Figure 5A. As shown in the figure, the liver morphology was markedly different in the HFD group compared to the Con group. A diffusely enlarged, light-colored liver with small yellowish-white granules on the surface was observed in the HFD group. Therefore, 12 weeks of HFD feeding dramatically altered the size and color of the liver compared to that of the Con group. However, LPQ1 and orlistat treatment effectively modified the liver morphology, with LPQ1 treatment markedly decreasing the liver size while improving the liver color. Moreover, LPQ1 treatment was more effective in restoring the liver morphology of HFD-induced mice than orlistat. Similarly, HFD feeding for 12 weeks led to an increase in the size of the epididymal tissues (Fig. 5A), while LPQ1 and orlistat treatment effectively decreased the size of the epididymal adipose tissues. Consequently, HFD feeding increased the liver weight in HFD-induced mice after 12 weeks of the treatment period. Interestingly, LPQ1 and orlistat administration dramatically decreased the liver weight of HFD-induced mice. The liver weight of LP-L, LP-H and orlistat reduced to 0.896 g (*p* < 0.0001), 0.941 g (*p* < 0.001) and 1.059 g (*p* < 0.05), respectively compared to that of the HFD group (1.284 g) (Fig. 5B). Therefore, LPQ1 treatment is effective in modulating the HFD-induced lipid accumulation in the liver. Similarly, less accumulation of epididymal adipose tissues (EAT), subcutaneous adipose tissues (SAT), visceral adipose tissues (VAT) and brown adipose tissues (BAT) were reported with LPQ1 treatment (Fig. 5C-5J). EAT accumulation in LP-L, LP-H and Orlistat groups decreased to 1.568 g, 1.265 g (*p* < 0.001) and 1.582 g, respectively compared to that of HFD (2.028 g) (Fig. 5C). Similarly, SAT weight also decreased with LPQ1 treatment (Fig. 5D). As shown in Fig. 5E VAT accumulation in LP-L, LP-H and Orlistat groups was reduced to 0.887 g (*p* < 0.001), 0.657 g (*p* < 0.0001) and 0.692 g (*p* < 0.0001), respectively compared to that of HFD the group (1.371 g). Subsequently, LPQ1 treatment markedly decreased the BAT accumulation (Fig. 5F). Therefore, LPQ1 administration effectively modulates the HFD-induced lipid accumulation in the liver and adipose tissues.

### LPQ1 Modulates Fatty Acid Metabolism and Liver Function in HFD-Induced Mice

**Fig. 6:**
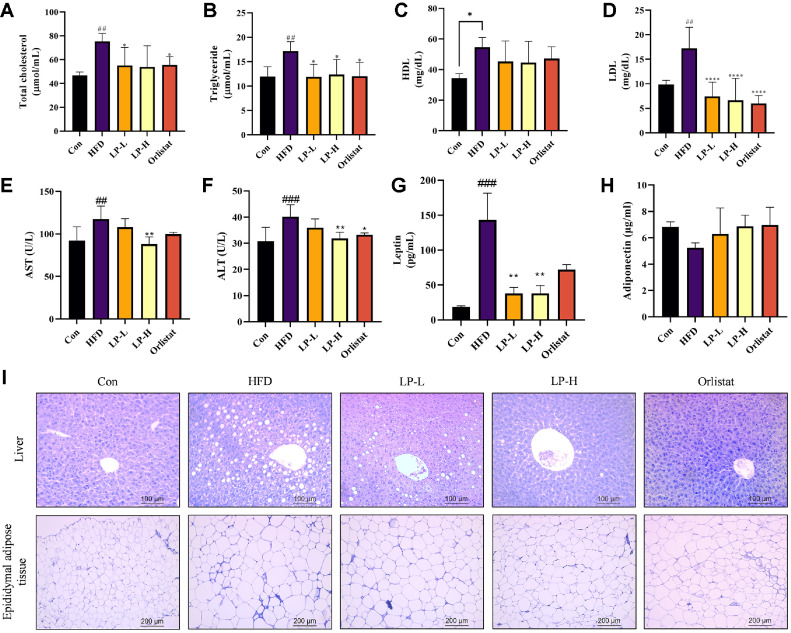
Oral administration of LPQ1 improved serum biochemical parameters and architecture of liver and epididymal adipose tissues in HFD-induced mice. Serum (**A**) Total cholesterol, (**B**) Triglycerides, (**C**) High-density lipoprotein (HDL) cholesterol, (**D**) Low-density lipoprotein (LDL) cholesterol, (**E**) Aspartate transaminase (AST), (**F**) Alanine transaminase (ALT), (**G**) Leptin and (**H**) Adiponectin levels of each treatment group. (**I**) H&E staining of liver and epididymal adipose tissues. C57BL/6 mice were fed with normal chow diet or HFD for 12 weeks and treated with LPQ1 or orlistat in last 4 weeks. After 12 weeks of experimental period all mice were sacrificed over anaesthesia and blood samples were collected and sera were separated to analyze biochemical parameters. Liver samples were collected and fixed in 10% formaldehyde for histological analysis. Con: control group; HFD: high-fat-diet group; LP-L: low dose LPQ1 treatment group; LP-H: high dose LPQ1 treatment group; Orlistat: positive control group. The results expressed as mean ± SD and the significant values are denoted based on the Dunnett's multiple comparisons test. *p < 0.05, **p < 0.01 and ****p < 0.0001 compared to HFD group. ## *p* < 0.01 and ### *p* < 0.001 compared to control.

HFD-induced mice were treated with LPQ1 or orlistat for a 4-week period and serum TC, TG, HDL-C, LDL-C, aspartate transaminase (AST), alanine transaminase (ALT), leptin and adiponectin levels were determined to investigate the modulating effect of LPQ1 on fatty acid metabolism. These serum biochemical parameters are displayed in Figure 6. Serum TC and TG levels were significantly elevated after 12 weeks of HFD feeding compared to the Con group proving that HFD-induced mice developed obvious hyperlipidemia and fatty liver. As shown in Fig. 6A and 6B LPQ1 treatment significantly (*p* < 0.05) decreased the elevated levels of serum TC and TG. Concomitantly, LPQ1 administration significantly (*p* < 0.0001) reduced the HFD-induced elevated levels of serum LDL-C (Fig. 6D). However, LPQ1 had no significant effect on serum HDL-C (Fig. 6C). Concurrently, LPQ1 modulates the liver function by altering the serum AST and ALT levels. LPQ1 administration significantly (*p* < 0.01) reduced the serum AST and ALT levels (Fig. 6E and 6F). AST and ALT are specific aminotransferases which increase with liver damage. Therefore, LPQ1 administration exhibits ameliorative effects against liver damage. Moreover, LPQ1 administration significantly (*p* < 0.01) reduced elevated levels of serum leptin levels in HFD-induced mice. However, the effect of LPQ1 on serum adiponectin levels was insignificant (Fig. 6E and 6H).

The effect of LPQ1 on lipid accumulation in the liver and epididymal adipose tissues was assessed using H&E staining and displayed in Fig. 6I. H&E staining revealed significant differences between the Con and HFD groups. The Con group exhibited intact lobular architecture with a clear central vein and radiation line, while the cell cords of the Con group were neatly arranged. In contrast, HFD group showed a large number of fat vacuoles that damaged the lobular architecture. Additionally, the radial arrangement of hepatocyte plates observed in the Con group was absent in the HFD group. However, the administration of LPQ1 significantly improved the lobular architecture. Furthermore, the large number of fat vacuoles observed in the HFD group gradually decreased with increasing doses of LPQ1.

To further investigate the effect of LPQ1 on histopathological changes in HFD-induced mice, epididymal adipose tissue sections were stained with H&E. As shown in Figure 6I HFD feeding significantly increased the thickness of fat pads and the mean diameter of adipocytes. However, the administration of LPQ1 effectively ameliorated the HFD-induced hypertrophic changes of abdominal fat pad. Subsequently LPQ1 administration decreased the thickness of fat pad and the mean diameter of adipocytes. This was more pronounced with high doses of LPQ1. Therefore, LPQ1 administration is effective in alleviating the HFD-induced lipid accumulation.

### LPQ1 Modulates Lipid Metabolism and Inflammation Related Markers in HFD-Induced Mice

**Fig. 7:**
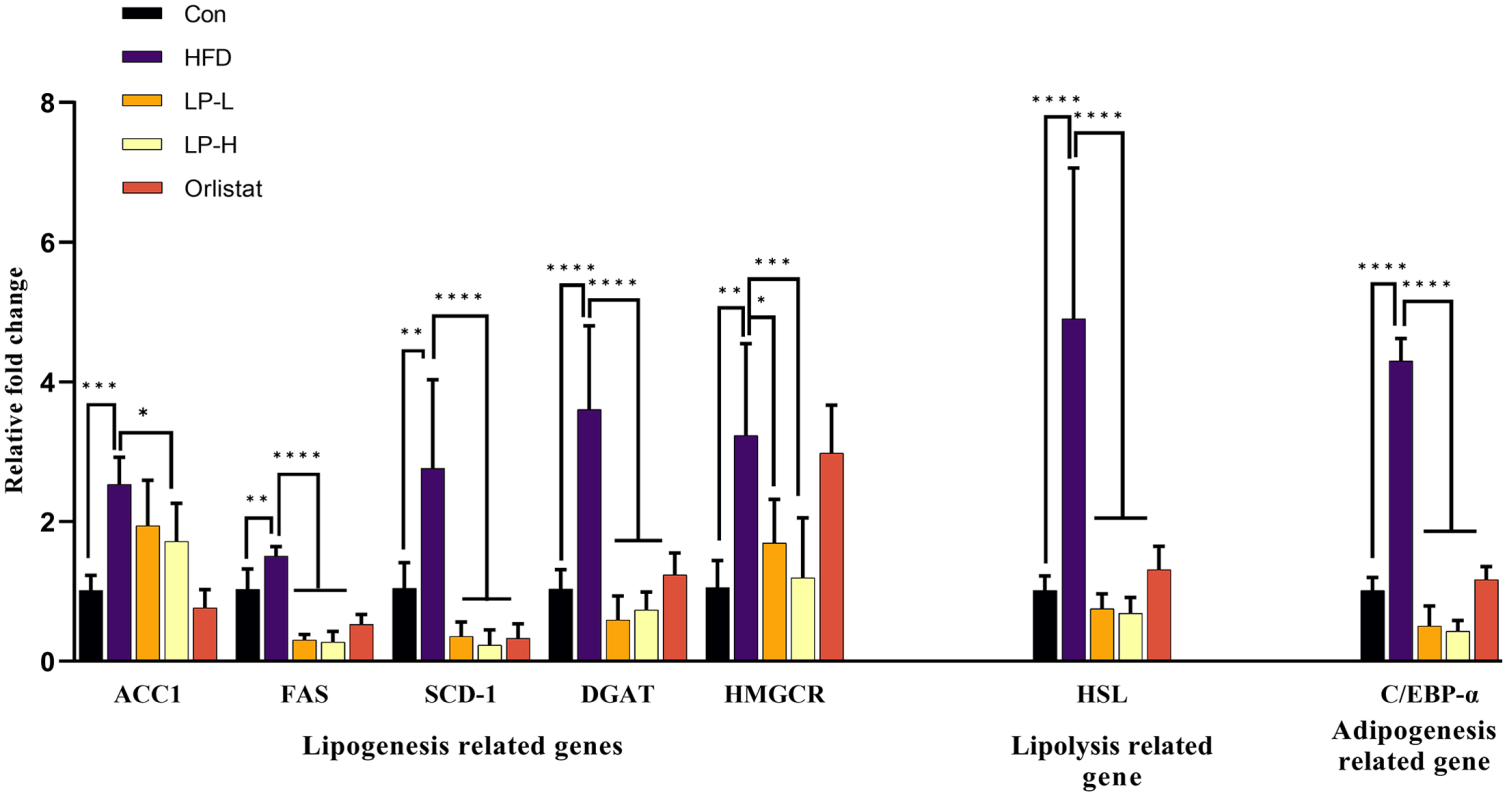
Oral administration of LPQ1 ameliorates lipogenesis, lipolysis and adipogenesis related hepatic gene expression. mRNA expression of ACC1, FAS, SCD-1, DGAT, HMGCR, HSL and C/EBP-α were determined by qPCR and normalized to GAPDH as an internal control. Con: control group; HFD: high-fat-diet group; LP-L: low dose LPQ1 treatment group; LP-H: high dose LPQ1 treatment group; Orlistat: positive control group. The results ex-pressed as mean ± SD and the significant values are denoted based on the Dunnett's multiple comparisons test. *p < 0.05, **p < 0.01, ***p < 0.001 and ****p < 0.0001.

The current study determined mRNA and protein expression levels of liver tissues of HFD-induced mice to confirm the ameliorative effect of LPQ1 on hepatic lipid accumulation. The mRNA expression levels, including ACC1, FAS, SCD-1, PPAR-γ, DGAT, HMGCR, HSL and C/EBP-α for lipid metabolism-related genes were assessed considering the related pathways pinpointed in the enrichment analysis results explained in section 3.1. As shown in Fig. 7 lipogenesis related genes: ACC1, FAS, SCD-1, DGAT and HMGCR were markedly elevated in the HFD group. However, LPQ1 treatment alleviated the overexpressed ACC1, FAS, SCD-1, DGAT and HMGCR genes. Concurrently, the lipolysis related gene, HSL and the adipogenesis related gene, C/EBP-α also significantly elevated in the HFD group. Similarly, LPQ1 administration significantly decreased the elevated levels of HSL and C/EBP-α mRNA expression. Therefore, LPQ1 administration effectively ameliorates hepatic lipid accumulation via its’ modulating effect on hepatic lipid metabolism.

**Fig. 8:**
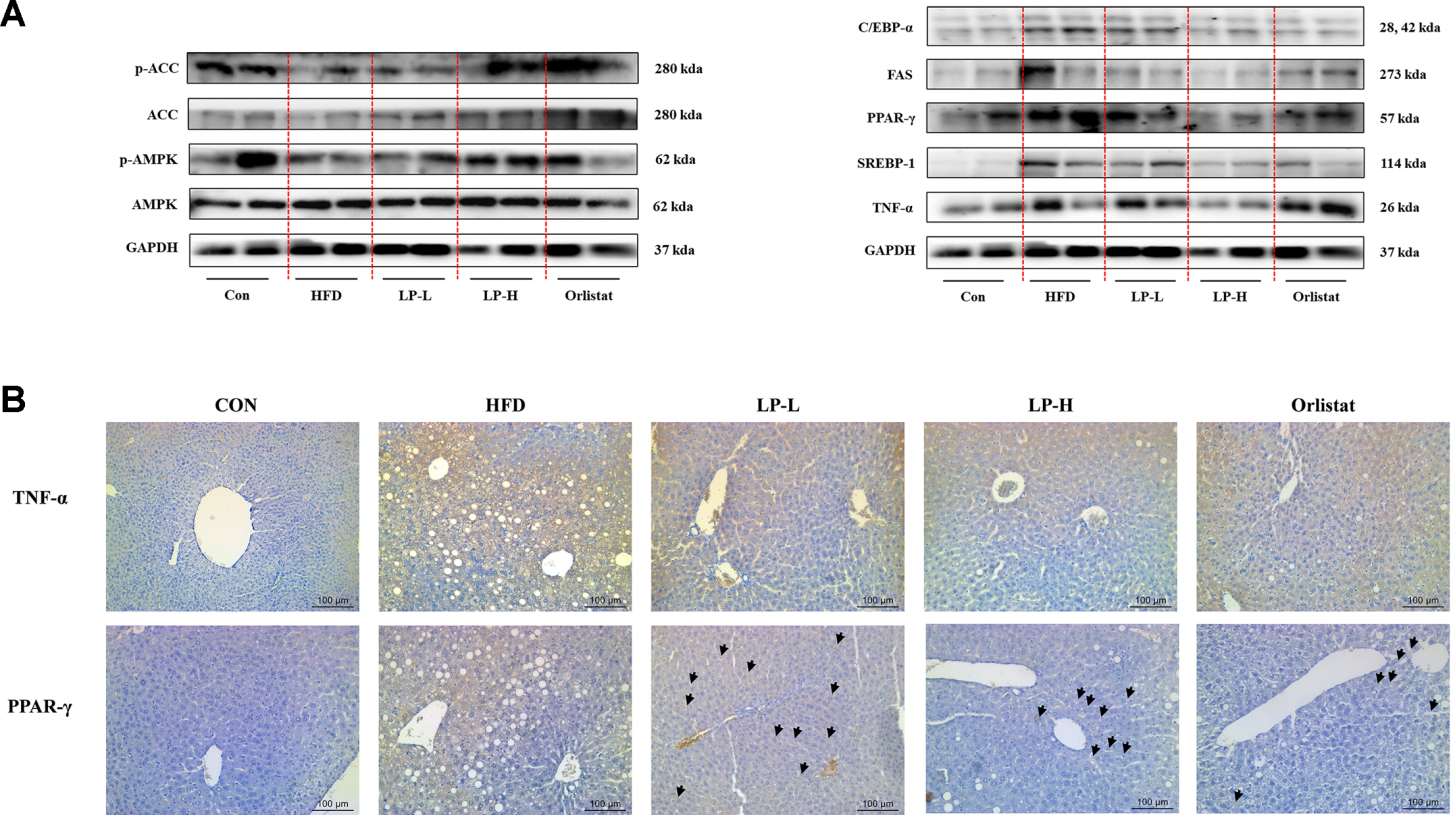
Oral administration of LPQ1 ameliorates lipid metabolism and inflammation related protein expression. (**A**) Representative images of lipid metabolism and inflammation related protein expression including p-AMPK, p-ACC, SREBP-1c, PPAR-γ, FAS, C/EBP-α and TNF-α. GAPDH was used as internal standard. (**B**) Immunohistochemical staining of TNF-α and PPAR-γ expression in liver sections of each group. Con: control group; HFD: high-fat-diet group; LP-L: low dose LPQ1 treatment group; LP-H: high dose LPQ1 treatment group; Orlistat: positive control group.

The network pharmacology analysis highlighted major biological processes associated with paraprobiotics in regulating lipid metabolism. To validate these findings, the current study determined the *in vivo* protein expression levels and Western blot results shown in Fig. 8A. These protein expression results displayed overexpression of the inflammatory marker, TNF-α in the HFD group. However, LPQ1 administration downregulated the overexpressed TNF-α. Therefore, LPQ1 is effective in reducing the inflammatory responses associated with fat deposition in the liver. Concurrently, LPQ1 administration dramatically decreased the overexpressed PPAR-γ protein levels in HFD-induced mice. PPAR-γ modulates the array of markers associated with inflammation, lipid uptake and storage [25, 26]. Therefore, PPAR-γ identified as one of the important markers linked with lipid metabolism. Notably, LPQ1 treatment downregulated the expression of FAS, a pivotal protein for lipid synthesis. Furthermore, LPQ1 administration suppressed the protein expression level of SREBP-1c compared to those in the HFD group. Similarly, LPQ1 administration markedly reduced the protein expression levels of C/EBP-α. On the contrary, LPQ1 administration promotes the expression of p-AMPK and p-ACC proteins in HFD-induced mice. Therefore, LPQ1 administration effectively activates AMPK and its downstream targets.

The current study further confirmed the network pharmacological results by immunostaining. The immunostaining results showed that LPQ1 administration dramatically decreased the TNF-α and PPAR-γ expression in liver tissues (Fig. 8B). Subsequently, immunostaining results further confirm the findings of network pharmacological analysis by proving the regulatory effect of LPQ1 on lipid metabolism via modulating the expression of TNF- α and PPAR-γ.

## Discussion

Obesity, NAFLD and diabetes are major worldwide health concerns that are highly linked with dysregulation of lipid metabolism [27-30]. Recently, probiotics have been identified as a potential intervention for the prevention and treatment of lipid metabolism disorders [31]. However, the specific effect of paraprobiotics on lipid metabolism disorders has not been fully explored regardless of the recent popularity of paraprobiotics as a potential functional ingredient. Therefore, the current study identified potential biological processes associated with paraprobiotics: LPQ1 related to lipid metabolism by network pharmacological analysis and validated those findings with *in vitro* and *in vivo* experiments. Subsequently, the current study provides broad insight into the specific effect of paraprobiotics, LPQ1, on lipid metabolism by integrating network pharmacology with *in vitro* and *in vivo* studies.

Currently, there is an emerging trend towards paraprobiotics as they exert similar beneficial effects to live probiotics. On the other hand, paraprobiotics provide a solution to probiotics-associated concerns. They have several positive characteristics over probiotics including longer shelf life, clear chemical structures and safety dose parameters apart from the beneficial health effects such as anti-obesity, anti-tumor, immunomodulation and preservation of the epithelial barrier [18, 20, 32]. Studies have suggested that the exerted beneficial effect of paraprobiotics is derived from the cell wall components and secretory metabolites where peptidoglycan, lipopolysaccharide, teichoic acid and cell wall-bound proteins play a crucial role. The high affinity of these isolated components to adhere to intestinal cells is higher than that of intact probiotic cells. This may be responsible for the exhibited health properties of paraprobiotics. Furthermore, paraprobiotics are effective through epithelial cells and mucus layers via contact with bioactive components [20]. Generally, the cell wall of *Lactobacilli* comprises a thick layer of peptidoglycan. Thus, this has been suggested as one of the major components of paraprobiotics that contribute to their health beneficial properties. These components are thought to exert different but complementary effects on host signaling pathways. Plethora of studies have proven the anti-inflammatory and immunomodulatory effects of peptidoglycan. Peptidoglycan has been reported to suppress interleukin-12 (IL-12) via Toll-like receptor 2 (TLR2), and by interacting with TLR2 on intestinal epithelial and immune cells, it initiates a MyD88-dependent cascade that modulates downstream regulatory factors including NF-κB and AMPK [18]. Activation of AMPK leads to the inhibition of adipogenic transcription factors such as PPAR-γ and SREBP1-c, which in turn reduces adipogenesis and the production of proinflammatory cytokines such as TNF-α. Lipoteichoic acid further enhances anti-inflammatory responses through TLR2-mediated pathways, thereby attenuating excessive inflammatory signaling [33]. In addition, secreted metabolites, especially short-chain fatty acids, directly stimulate AMPK activity, promoting fatty acid oxidation while suppressing the expression of key lipogenic enzymes. These mechanisms combine to form a multi-target regulatory network that reduces hepatic lipid synthesis and attenuates inflammatory responses. Such regulation may have a significant effect in alleviating metabolic dysregulation observed in NAFLD and obesity-related diseases. These reported findings are in line with the networks analysis of the current study, where the toll-like receptor signaling pathway has been identified as one of the major pathways in the KEGG enrichment analysis.

Network pharmacology analysis identified TNF-α, IL-18, IL-1β and PPAR-γ as major targets of LPQ1 in lipid metabolism disorders. Additionally, the inflammation-related pathway was the most common pathway shared in enrichment analysis. Therefore, the beneficial effect of LPQ1 on lipid metabolism might be exerted through this inflammation-related pathway. To validate these results, we used *in vitro* and HFD-induced *in vivo* models. The findings of the *in vitro* and *in vivo* studies confirmed that LPQ1 reduced the overexpressed levels of TNF-α and PPAR-γ. Oxidative stress and inflammation play a major role in the progression and aggravation of NAFLD-like lipid metabolism disorders. Liver inflammation eventually leads to the progression of NAFLD to NASH activating proinflammatory cytokines and adipokines. In the initial stage, the role of lymphocytes is crucial in aggravating liver inflammation, which triggers the generation of IL-17, a Th17-derived cytokine, and TNF-α. Elevated levels of these molecules are featured in the liver of NAFLD patients [34]. Network pharmacology analysis of the current study identified IL-17 signaling, toll-like receptor signaling and TNFα signaling pathways as major targets of paraprobiotics in lipid metabolism proving the potential of LPQ1 in alleviating liver inflammation. Similar results were reported in a previous study where the administration of heat-inactivated *Lactobacillus rhamnosus* prevents diet-induced liver inflammation in rats [35]. Another, study also suggested that suppression of TNF-α might be the potential mechanism of heat-killed *L. brevis* SBL88 on exerted inhibitory effect of NAFLD [36].

The current study first validated the results of network pharmacology analysis *in vitro* with HepG2, FL83B and 3T3-L1 cells. HepG2 and FL83B cells were grown in a palmitate-rich medium to induce lipid accumulation and subsequently treated with LPQ1. Results of the current study revealed that LPQ1 treatment dose-dependently decreased the PA-induced lipid accumulation in HepG2 and FL83B. A previous study also reported similar results, where heat-killed *Enterococcus faecalis* inhibits lipid accumulation in FL83B cells [37]. Results of the current study also confirmed the ameliorative effect of LPQ1 on lipid accumulation in 3T3-L1 adipocytes. Concurrent with the present study, a recent study has reported the inhibitory effect of heat-killed *E. faecalis* on lipid accumulation in 3T3-L1 adipocytes. The reported study confirmed that treatment of heat-killed *E. faecalis* decreased the level of ORO staining in concentration dependent manner [38]. Similar results were reported with previous study where heat-killed *L. plantarum* K8 markedly inhibit the intracellular lipid accumulation in 3T3-L1 adipocytes. Moreover, re-ported study further determined the mRNA levels of transcription factors PPAR-γ and FAS and reported similar results with the current study where treatment of heat-killed *L. plantarum* K8 dose-dependently decreased the elevated levels of PPAR-γ and FAS [39].

The present study determined the ameliorative effect of LPQ1 on lipid metabolism *in vivo* and confirmed that oral administration of LPQ1 markedly decreased the serum total cholesterol, triglyceride and LDL cholesterol levels while downregulating the lipid metabolism related gene and protein expression. Similar results were reported in a previous study where treatment of heat-killed *L. reuteri* GMNL-263 attenuated the obesity-induced metabolic abnormalities and hepatic steatosis formation [40]. Another study has reported that administration of heat-killed *E. faecalis* decreased the cholesterol levels while inhibiting the insulin signaling pathways in HFD-induced obese rats [41]. A study also confirmed the anti-obesity effect of heat-killed lactic acid bacteria on high-fat and high-fructose diet fed mice. The study further suggested that heat-killed lactic acid bacteria demonstrated the modulating effect on adipogenesis through the regulation of PPAR-γ expression via toll-like receptor 4 (TLR4) signaling [42]. Similar to the reported study, findings of the current study also confirmed the modulating effect of LPQ1 on PPAR-γ expression. A recent study also reported that heat-killed *L. plantarum*, LPHK exhibited greater potential in decreasing HFD-induced body weight gain, liver weight and adipose tissue weight gain while lowering the plasma total-cholesterol, triglyceride and LDL-cholesterol concentrations [43].

Fatty liver is identified as one of the critical conditions linked with lipid metabolism disorders. In this process, a number of signaling molecules activate the lipid metabolism-related transcription factors such as PPAR-γ, FAS, SREBP1-c and ACC [44]. PPAR-γ is involved in the regulation of adipogenesis, glucose homeostasis and inflammation. As reported in previous studies, hepatic PPAR-γ stimulates *de novo* lipogenesis, leading to fat accumulation [45]. Therefore, the expression of PPAR-γ is featured in fatty liver. Subsequently, activation of PPAR-γ induces NAFLD development in animal models and disease onset can be prevented by the deletion of PPAR-γ [46]. Therefore, PPAR-γ is identified as one of the major transcription factors in lipid metabolism. Interestingly, network pharmacology analysis of the current study has identified PPAR-γ as one of the top ten hub genes associated with paraprobiotics, proving the effectiveness of LPQ1 in ameliorating lipid accumulation by modulating the expression of PPAR-γ. Concurrently, *in vitro* and *in vivo* results of the current study also confirmed the effectiveness of LPQ1 treatment in downregulating the overexpression of PPAR-γ. SREBP is another important transcription factor that is linked to fatty liver. SREBP-1c promotes the upregulation of genes involved in de novo lipogenesis following activation by saturated fatty acids [45]. A previous study reported that deletion of SREBP-1c results in a 50% reduction in fatty acid synthesis. Accordingly, SREBP-1c promotes the expression of hepatic lipogenesis genes such as ACC, FAS and stearoyl-CoA desaturase-1 (SCD1), while PPAR-γ mainly induces the proteins linked to fatty acid uptake, binding, and transport. Moreover, studies have suggested that overexpressed liver PPAR-γ expression in obesity serves as a prolipogenic factor reinforcing lipogenic mechanisms linked to SREBP1-c induction [46]. Giving priority to this explanation, the current study determined the expression levels of PPAR-γ, SREBP1-c, ACC, FAS and SCD1 *in vitro* and *in vivo* and confirmed that LPQ1 downregulated the overexpressed PPAR-γ, SREBP1-c, ACC, FAS and SCD1. On the contrary, the current study also determined the expression level of TNF-α, an inflammatory response reported to play a crucial crosslinking role in the development of fatty liver. Studies have proven elevated inflammatory levels in mice fed with HFD, where the IκBα-NF-κB pathway was overactivated, leading to increased mRNA expression levels of TNF-α, IL-6 and IL-1β [47, 48]. The results of the current study revealed that protein expression levels of TNF-α were markedly increased in the HFD group compared to that of the control group, and as expected, LPQ1 treatment effectively decreased the overexpressed TNF-α. The current study further determined the relative mRNA expression level of TNF-α in 3T3-L1 adipocytes and confirmed that LPQ1 treatment dose dependently decreased the elevated levels of TNF-α. These findings were perfectly matched with the network pharmacology analysis where TNF-α, IL-18 and IL-1β were identified as top hub genes associated with the ameliorative effect of paraprobiotics on lipid metabolism. Furthermore, several studies have reported the association between obesity and inflammatory response.

The findings of the current study suggest that the modulating effect of LPQ1 on lipid metabolism may be associated with the regulation of lipid metabolism-related transcription factors, including PPAR-γ, SREBP1-c, and inflammation-related transcription factors. These findings support the potential of LPQ1 as a promising candidate for the treatment of NAFLD and obesity‐related lipid metabolism disorders. However, one notable limitation of this study is that it exclusively investigated the effects of heat-killed *L. plantarum* Q1 (LPQ1) on lipid accumulation in both *in vitro* and *in vivo* models, without direct comparison to its live counterpart. Although the use of heat-killed bacteria offers advantages in terms of safety, stability, and shelf life, the absence of comparative data limits our ability to determine whether the observed effects are unique to the heat-killed form or shared with live bacteria. Given that live probiotics may exert distinct biological activities, particularly through colonization and metabolite production, future studies are needed to directly compare the efficacy and mechanisms of live and heat-killed LPQ1.

## Conclusion

The current study employed an integration approach to confirm the ameliorative effect of LPQ1 on lipid accumulation where the study combined network pharmacology with *in vitro* and *in vivo* experiments. As revealed by the findings of network pharmacology, targets of paraprobiotics were highly enriched with lipid metabolism and inflammation-related signaling pathways. In line with the network pharmacological analysis, the findings of *in vitro* and *in vivo* experiments also confirmed that LPQ1 is effective in improving lipid accumulation and inflammation in HFD-induced mice and PA-induced HepG2 cells, as well as 3T3-L1 cells. LPQ1 modulated hepatic lipid synthesis through its regulatory effect on transcription factors PPAR-γ, SREBP1-c, ACC, FAS and SCD1 while also effectively alleviating inflammation by regulating the transcription factor TNF-α. However, the specific mechanism involved in the ameliorative effect of LPQ1 on lipid accumulation and compare the activity of live and heat-killed LPQ1 need to be further explored.

## Supplemental Materials

Supplementary data for this paper are available on-line only at http://jmb.or.kr.
