# Supplementary material for: Ameliorative Effect of Heat-Killed Lactiplantibacillus plantarum Q1 (LPQ1) on Lipid Accumulation: Integration of Network Pharmacology with In Vitro and In Vivo Experiments
Source: J Microbiol Biotechnol. 2025 Jul 29;35:e2503038. doi: 10.4014/jmb.2503.03038 (PMC12325000; doi:10.4014/jmb.2503.03038)
Supplement: Supplementary file 1 [file jmb-35-e2503038-supple.pdf]

J. Microbiol. Biotechnol. 2025. 35: e2503038
https://doi.org/10.4014/jmb.2503.03038

Supplementary Tables

Table S1. Primer sequences of the genes used in RT-PCR analysis.

Gene name		Sequence
SREBP-1c	Forward	5-GGC TCC TGG CTA CAG CTT CT-3
	Reverse	5-CAG CCA GTG GAT CAC CAC A-3
FAS	Forward	5-TAT GCT TCT TCG TGC AGC AGT-3
	Reverse	5-GCT GCC ACA CGC TCC TCT AG-3
ACC1	Forward	5-CAG AAG TGA CAG ACT ACA GG-3
	Reverse	5-ATC CAT GGC TTC CAG GAG TA-3
GAPDH	Forward	5-CCC CTG GCC AAG GTC ATC CAT GAC AAC TTT-3
	Reverse	5-GGC CAT GAG GTC CAC CAC CCT GTT GCT GTA-3

.

Table S2. Primer sequences of the genes used in RT-PCR analysis.

Gene name		Sequence
ACC1	Forward	5-GGG CTA CCT CTA ATG GTC TT-3
	Reverse	5-CTA CCT GAT GGT AAA TGG GA-3
FAS	Forward	5-CTT GGG TGC TGA CTA CAA CC-3
	Reverse	5-GCC CTC CCG TAC ACT CAC TC-3
SCD1	Forward	5-CGG AAA TGA ACG AGA GAA GG-3
	Reverse	5-CCG AAG AGG CAG GTG TAG AG-3
DGAT	Forward	5-CCT CAG CCT TCT TCC ATG AG-3
	Reverse	5-ACT GGG GCA TCG TAG TTG AG-3
HSL	Forward	5-GAA AGA CAA CGG ACA AAT CAC-3
	Reverse	5-GAA ACT GGC ACC CTT GAA-3
HMGCR	Forward	5-TTC TGC TCT TGA TTG ACC TTT C-3
	Reverse	5-TTT CCC TTA CTT CAT CCT GTG A-3
PPAR-γ	Forward	5-GAA AGA CAA CGG ACA AAT CAC-3
	Reverse	5-GAA ACT GGC ACC CTT GAA-3
FAS	Forward	5-CTT GGG TGC TGA CTA CAA CC-3
	Reverse	5-GCC CTC CCG TAC ACT CAC TC-3
TNF-α	Forward	5-AAG CCT GTA GCC CAC GTC GT-3
	Reverse	5-GGC ACC ACT AGT TGG TTG TC-3
GAPDH	Forward	5-GCA CAG TCA AGG CCG AGA AT-3
	Reverse	5-GCC TTC TCC ATG GTG GTG AA-3

Table S3. Corresponding targets related to major constituent of paraprobiotics.

Major constituent	Symbol
Peptidoglycan	TIRAP, TLR4, CXCR3, CXCL10, MYD88, TRAF6, TLR9, CD289, NOD1, RIPK2, TLR5, IRAK4, PHLPP2, PHLPP1, TLR3, NOD2, TLR6, TLR2, TLR7, MAPK3, MAPK1, TLR1, IL18, IL1B, TLR8, PLIN1, PPARG, MAPK8, PTGS2, IL8, IL6, IL10, TNF, CD83, SPG7, CCL20, TLR10, CSF2, MMP1, PGLYRP1, YME1L1, CXCR5, NLRP3, CD79A, GP5, LYZ, DCN, EIF4E, PGLYRP3, PGLYRP2, PPM1L, PGLYRP4, MEFV

Table S4. Differentially expressed genes common to obesity, NAFLD and lipid metabolism disorders.

Symbol
PPARG, LEP, ACACA, INS1, SOD1, FASN, SREBF1, CAT, ADIPOQ, CPT1A, IL1B, PPARA, GPX1, TNF, NOS2, RELA, TGFB1, GSR, FOS, CDKN1A, IL6, SOD2, IGF1, NFE2L2, NFKB1, BCL2, AKT1, MAPK3, INS, MAPK1, IL10, PTGS2, FABP4, HMOX1, CCL2, GSK3B, BAX, CD36, CASP3, VCAM1, PPARGC1A, DDIT3, FAS, PCK1, CCND1, CTNNB1, HMGCR, CEBPA, MMP2, TP53, ICAM1, SCARB1, MAPK8, PRKCA, ALB, INSR, ALPL, LDLR, GPT, TH, CREB1, CYCS, FSHB, NQO1, NFKBIA, CYP1A1, VEGFA, JUN, BDNF, LPL, CYP11A1, SLC2A4, ACOX1, SIRT1, IRS1, SQSTM1, IFNG, PIK3R1, ACTA2, BCL2L1, NR3C1, GJA1, AHR, CDH1, NPY, STAR, CCNB1, IGF1R, CYP17A1, GAPDH, ESR1, CASP9, STAT3, DNMT1, SPP1, EGR1, PNPLA2, LHB, SCD1, TIMP1, IDI1, AGT, COL1A1, APP, ACACB, CASP8, GCLC, CYP1B1, MAPK14, PARP1, CYP1A2, CYP3A4, MTOR, MAPK9, POMC, CEBPB, CDKN1B, MYC, SOX9, CAMK2A, HSPA5, STAT1, XDH, DUSP1, FOXO3, SREBF2, APOE, IL18, SGK1, CXCL8, CASP1, MPO, MMP9, PRL, EGFR, NR0B2, CRP, HSD3B1, TJP1, FOSL1, GOT1, CYP19A1, PLIN2, NOS3, CASP7, ABCA1, LDHA, PCNA, TRP53, ATF3, VIM, FN1, PKM, SLC2A2, SERPINE1, HMGCS1, G6PD, CTSD, ABCC2, CCND2, XBP1, CYP7A1, ATF6, TLR4, NTRK2, GSTP1, PTEN, KDR, NCF1, ABCG2, RELN, CCNA2, CDK1, PRKAA1, CLU, TRIB3, MET, IRS2, IGFBP1, GADD45A, MKI67, LIPE, SCD, IL4, TTR, CYP2E1, CXCL12, CCNE1, ALDOA, ANGPTL4, CXCL10, TNFRSF1A, FGF2, UCP2, EIF2AK3, OCLN, GFAP, GPX4, AKT2, SLC2A1, GPAM, MAP1LC3B, MMP7, CXCL1, PRLR, ACADM, PPARD, SFRP2, PDK4, NOX4, RPS6, ESR2, NR1I2, TXNIP, SOCS3, LCN2, NAMPT, CYBB, SLC17A8, CREBBP, ADIPOR2, GRIN1, RB1, NR1I3, CXCL5, JUNB, TOP2A, CD14, RXRA, ATP1A1, MAOB, AR, RUNX2, ABCB1, ERN1, HSD11B1, NR1D1, HIF1A, DIO2, ATG5, CYP2B10, PHGDH, ACLY, GCLM, ZBTB16, TSC22D3, TIMP2, ACHE, HSPA1B, TGFB2, SRC, IGFBP2, ATF4, CDK2, IL33, ID2, INSIG1, DPP4, CHEK2, DLK1, ACTA1, VNN1, SP1, LEPR, BMAL1, FBXO32, GCK, UBE2C, DDIT4, UGT1A1, MDM2, APOB, SC5D, CYP4A14, PDK1, CCN2, ACSL5, IL12B, ID3, MTTP, HSP90AA1, H2AX, TGFBR2, DCLK1, HNF4A, BAD, COMT, ALCAM, KLF4, B2M, GSTA1, ENO1, CCL3, MSMO1, CDH2, SMAD2, TGFB3, ASNS, CPE, LPIN1, MAOA, SMAD3, TNFRSF10B, ATG7, SOD3, BID, THBS1, SLC3A2, DNMT3A, APOA1, PLD1, S100A9, POR, ATP2A2, ACE, TSKU, FASLG, TIMP3, ITGB1, JAK2, CDK6, S100B, TFRC, GSTM2, IKBKB, FABP1, NGF, DGAT1, CBS, C3, CCND3, FGF21, CROT, NUPR1, TLR2, DGAT2, EFNA1, HSP90B1, HSP90AB1, JUND, TFAM, PRKCZ, BAK1, NLRP3, DUSP6, CYBA, PNPLA3, NR1H3, SQLE, CX3CL1, PRKCE, SNCA, EHHADH, IGFBP3, TXNRD1, CFB, ACSM3, BECN1, DHCR7, INHBB, DUSP5, CHUK, FABP3, NCOA1, GSTA4, CP, EDN1, FLT1, GPX2, ALDH1A1, FADS1, FDFT1, EGF, MFSD2A, BCL2L11, CLOCK, CHEK1, HP, VDAC1, RPS6KB1, ALDH9A1, CPT2, PER2, ABCC3, CREB3L2, SLC6A6, IL1RN, ITGAV, CFD, PDIA3, ANXA2, CDK4, IGFBP4, CEBPD, GSTM1, BNIP3, PCK2, BHMT, AFP, SOCS2, VLDLR, EIF2S1, ALDH3A2, HSD17B10,

CASP4, PTK2, CCL5, UHRF1, GLUL, GDF15, UCP1, CASP12, OGG1, ADGRE1, ABHD2, CD44, MVD, PPP1R3C, KEAP1, MLXIPL, CD68, TSC22D1, CAV1, PRDX2, CDKN2A, HSPB1, WNK2, GHR, PRKACA, CALR, INSIG2, PON1, HAVCR1, NRG1, CYP26B1, ACAD11, TDO2, GBP2, MT2A, EGR2, IGF2, SLC27A1, PER3, MMP3, BTG2, HSD11B2, PBK, EIF2A, MT1, GPER1, SLC27A2, EIF4EBP1, LCAT, SCP2, RAC1, CD86, GSTM3, NDRG1, DNAJB9, CIDEA, TF, MGLL, UCP3, DNMT3B, SKP2, ACAT2, SLC38A4, SYP, SLC7A11, TNFSF10, PRKAA2, PROM1, LRRN2, MAP2K4, PDGFRB, AREG, PLAUR, CXCR4, A2M, ANXA5, GLRX, SORCS1, SLC1A2, TPI1, SPTLC3, PDLIM1, MLPH, CYP7B1, TFAP2A, ABCC1, CDC20, CYP3A11, MME, RETN, TBC1D4, IGFBP5, ALDH2, ME1, ACADL, AGTR1A, OLFML1, MAP2K2, MAP1B, RBP4, ULK1, PRNP, MMP13, IL13, G6PC1, C4B, GAD1, MYLK, SLC6A4, AXIN2, PHB1, SLC1A3, ARNT, CPT1B, PDGFRA, IGF2R, PRKCD, BHLHE40, ARG1, FOXO1, IL17A, GPX3, MIF, F2R, PRDX1, FGFR4, MUC16, CCL7, HDAC1, ID1, SELE, GSTA2, NCAM1, AQP8, HSD17B4, HSPE1, IRF1, MCM2, MAP2, ZFP36, ABCG1, EP300, NR1H4, THRB, PSMB8, LIPG, MAP2K1, HSD3B2, MFN2, ANXA1, KRT18, VCAN, ERBB2, PTGS1, GSN, HMGB2, PIM1, PRKCB, PTPN1, GATA4, E2F1, AQP7, IVNS1ABP, DPP6, GSS, CCR7, SERPINF1, DBP, DLD, TGFBI, IL2, HSPD1, HSD17B1, ITIH2, CDKN1C, ITGA5, AKR1A1, ENC1, GGT1, NFATC1, ABCC4, GAL, BIRC5, ENPP2, PKLR, MBP, NTN1, FOXA2, SLCO1A1, EPHX2, ABCB11, GHRL, ATP7B, BMP7, ITGB3, MCM4, ACOT4, ALAD, FDPS, TPM1, FGFR1, HK2, HSPA2, LDHB, PCSK9, USP2, NR4A1, VDR, CRYAA, ASS1, DHRS9, NPAS2, GADD45G, ITPR1, ATP1B1, IFIT1, NRF1, TIPARP, XRCC1, GLB1, ELOVL6, NOTCH1, OGDH, APAF1, ADGRA1, NFIL3, LMNA, PITPNC1, COL5A2, TAT, CYP4A12A, HSPA9, CANX, KYNU, TNFRSF21, ABCB1B, ABCB4, JUP, GOT2, NR4A2, ANPEP, BACE1, GPD1, JAK1, MMP1, DIO1, TNFRSF11B, CCNA1, IL12A, CKB, CYP26A1, F3, MAPT, CXCL2, COL3A1, LSS, TNFRSF1B, FADS2, IRF3, CRY1, ITPR3, PGF, DNM1L, IFIT3, MT1A, SCNN1A, LYZ, HGF, FBP1, TSPO, PDGFRL, IFIT2, CD9, COL1A2, ABCB1A, SERPINB2, SLC22A1, CD40, TAGLN, DECR1, HSD17B2, TUBB3, PMVK, GADD45B, AQP1, TAC1, TRAF6, HPCAL1, ACSL4, CDC25B, MAP2K6, SLC11A2, GSTT1, CST3, COL6A3, SMC4, ABI3BP, CDKN2B, ACADVL, ITGA2, IRF7, GSTO1, MT2, CLDN1, SERPINC1, ACOT1, ABCD2, ITGAM, HAMP, POU5F1, PAX6, SCAP, DNAJC10, FLG, NFE2L1, PXN, AK4, BCO1, DLG2, NFIX, AKR1C6, BRCA1, TMPRSS2, HMGA1, NEK2, NME1, SLC40A1, CTH, CTSB, KRT5, SLC7A5, PLA2G12B, ORM1, NOS1, AGTR2, ANP32A, SLC25A1, RASA3, CSF1R, EPB41L3, SLC12A2, BBC3, BMPER, MYO5A, PDCD4, SLC22A5, CAMK2D, SMARCA2, HERPUD1, PINK1, ERBB3, SELP, DEPP1, LGALS3BP, SLC22A7, FOSB, HSPH1, IQGAP2, BCL6, EPCAM, FBXO30, MRC1, MX1, PTGDS, TNFRSF9, COX1, PSMC5, SLCO2A1, SORBS1, BMP2, MGMT, BCHE, EIF5, GALNT10, DKK1, KRT8, S100A8, CAR3, SFRP1, HACL1, TOMM20, DCN, EZR, NRP1, HK1, IL1A, SULT1A1, ADIPOR1, BBOX1, HSPA1L, SYN1, GMFB, BAG3, CD74, CENPF, EPHX1, PPP1R15A, EEF2, SELENOP, SH3BGRL2, CRELD2, NFKB2, RARRES2, TGFA, RAF1, LIFR, WT1, MX2, PABPC1L, KIF11, GPD2, GALNT2, CTNNAL1, MYD88, OPA1, PTPRF, VASP, CYB5B,

MFAP4, CFTR, ATM, CXCL9, GPNMB, LRP4, MUC2, NR2F2, CTSL, FKBP5, SESN2, SLC2A3, NREP, COL27A1, ACE2, ALAS1, CYP2C29, KRT17, MSR1, PPARGC1B, TGFBR1, RTP4, HBEGF, HES1, COL4A1, PNRC1, PENK, STMN1, DNM1, RUNX1T1, COX4I1, ARG2, PEG3, PYCARD, S100A6, NDUFS1, SLC39A8, CHPF, TFF1, ECH1, HYOU1, DERL3, HIF3A, PDGFB, THRA, NR1D2, COTL1, IGFBP7, MAFF, FAM163B, GLS, PGK1, CSRP3, ATL2, HDC, MDK, SRRM2, FMO5, CYP27A1, AKR1B1, ARPC1B, EGLN1, TRPV6, MFAP5, PRSS23, VCL, CELF3, OGA, HSPA1A, HADHA, IDH2, CDH13, LY6E, AKAP12, UNG, SCD2, FMO1, MALL, EMP1, GCG, IRAK1, F2, SLC9A1, PEG10, ETS2, SLCO1A4, CBX6, GABRP, HSPA12A, UBR4, AOC1, ABLIM1, TKT, CDC25C, RGS2, EIF2S2, FOXA1, VTN, CDKN2C, NEDD9, UBE2L6, VWF, SRSF7, TNC, G0S2, PTGES, CLDN4, PRKAR2B, GC, PLEC, FHL2, FTH1, AARS1, AQP9, KNG1, COX6A2, ADAMTS1, CCNB2, FSIP1, SLC5A1, ACAA2, LIPC, MMP12, ACTB, ANTXR1, LGALS1, NPPA, RAPH1, CYP4A10, PLAU, HMGB1, HPGD, LRP1, CIDEC, LBP, SESN1, VCP, SIRT3, SMOX, NFATC2, GNAI2, F7, CCNG2, HADH, PMP22, FLNB, RIPK1, IER3, GALNT16, NCF4, PRDM16, S100A14, ODC1, FBLN2, GALE, SLC25A10, CYP8B1, PGD, MAP1LC3A, MAP3K5, ADGRB1, DIRAS2, LOX, ITSN1, AQP4, EIF4EBP2, SIK1, TTK, MRAP, HSPA8, SMAD1, SP7, SFPQ, SPHK1, SLCO2B1, GLO1, NFKBIB, SHH, RRBP1, SH3KBP1, CSF2, CFLAR, CD52, NTRK1, SMAD4, FASL, ITGA3, PYGM, STAT5B, IL1R1, F5, PECAM1, PLCB1, RAN, TNFAIP3, EGR3, NUF2, XRCC5, IFIH1, OSMR, SPARC, WIPI1, SLC16A3, SLC1A5, UGDH, KMT2A, MAPK13, MGST1, PSAT1, RRM2, HRAS, BCL3, LITAF, ARIH2, NRIP1, SERPING1, ADORA1, PDX1, WNT7A, EGLN3, TAP1, GLIPR1, HNMT, PARP14, BDH1, ROCK1, TJP2, EIF4E, SRXN1, GALNT6, TMTC2, ANO1, CD63, DST, DSCAML1, GNAT2, FADD, ARHGDIA, CTNNA1, CYP4A1, FMO2, KIF20B, NCOA4, PGM1, PTMS, ENPP1, FMO3, EFNA5, TECR, MYH7, NANOG, ABCC5, ATP5F1B, TPM3, DDR1, GPI, GSK3A, SUCLG2, ADAM9, EPSTI1, GDF10, RAMP2, ALDH3A1, IL1RL1, STIP1, ERO1A, NCL, PHLDA2, SPC25, H19, STAT2, PRKAG2, AURKA, UGT1A9, PFKFB3, CCN1, PI16, MCM5, SELENBP1, SDC4, CD163, RAMP1, ANKS1B, ABCG8, COL12A1, PLTP, CCNG1, BCAR3, PSMB10, DAPK1, KLF5, DNAJC3, MGST3, SLC16A1, GPRIN1, FABP7, DUSP4, WNT5A, WWTR1, BGN, PSMC4, GH1, HSF1, MAFA, MYH1, MKNK2, CHKA, RBM3, RTN4, TUBB2B, ENO3, FOXM1, IL1R2, RGS4, SORL1, SPARCL1, AHCY, SLC25A20, BST2, CSRP2, CYP2B9, PLXDC2, CYP24A1, LEF1, NT5E, RSAD2, TCF7L2, FAM13A, HMOX2, RAD51, QKI, SCARA5, ARC, APOA4, ERRFI1, HSPG2, PGAM1, FGG, ANGPT1, LAMB1, SDHB, TYMS, H2-AB1, NR4A3, RBP1, COL14A1, RARB, OXR1, PYY, ADORA2A, GSTK1, CSF1, MCM6, NPPB, PHLDA1, RUNX1, TERT, NT5DC2, HRG, HMGCS2, IFI44, P4HA1, ACOT2, RND3, PPM1B, ALDOB, FEN1, PYGL, RGS16, CDKN3, KIF20A, NPDC1, SETD7, CELSR3, ASPH, CNN1, NPC2, PDK2, NPM1, PDE4B, RORA, STC1, AMPD3, BSG, DBN1, CD34, RABGAP1L, CLSTN3, SGK3, MCL1, FST, NCF2, TXN1, EFNB2, ST3GAL5, ACAT1, CDC42, PLOD3, GSDMD, MT3, C8G, FLNA, ABLIM3, FECH, SLC2A9, GAS6, KIF23, MCM7, PSMB1, SLC51B, TCF4, CYP2C9, GLUD1, NOTCH2, PLP1, RARA, GDA, CREM, PEBP1, WNT4,

	S100A11, GPCPD1, SIAH2, UCK2, AQP3, SULT2A1, CKS1B, FSTL1, NR2F1, SMAD7, REV1, COL2A1, AMIGO2, HAO2, MYH10, TNFRSF25, FOSL2, ISG15, PGS1, KRT20, SLC22A2, ADAMTS2, GFOD1, COL18A1, OXTR, PDHB, SYNPO, COL6A1, CRAT, NCAPG, PLS3, CAPN2, MVP, BCL11A, PAFAH1B1, CADPS2, GSTM6, SYVN1, WEE1, CNR1, FAT1, SLC4A4, PLAT, APLP2, ITIH3, MDH1, ENDOD1, RHBDF1, SACS, RPL29, CCNT1, GPT2, CTSS, PRDX6, SERPINE2, GZMB, PPBP, PZP, ACO1, LAMA5, PIK3R3, ABCG5, RHOB, ACBD5, KIF5B, CLMN, RIDA
